# Influence of Trunk Function on Assisted Gait and Independence in Patients With Acute Stroke

**DOI:** 10.1155/srat/9436252

**Published:** 2026-04-20

**Authors:** Masahiro Ishiwatari, Akihiro Ogawa, Masato Hayakawa, Satoshi Kido

**Affiliations:** ^1^ Faculty of Health Sciences, Uekusa Gakuen University, Chiba, Japan, uekusa.ac.jp; ^2^ Faculty of Social Work Studies Department of Physical Therapy, Josai International University, Chiba, Japan, jiu.ac.jp; ^3^ Department of Rehabilitation, Shioda Hospital, Chiba, Japan; ^4^ Graduate Course of Health and Social Services, Saitama Prefectural University, Saitama, Japan, spu.ac.jp

**Keywords:** acute stroke, gait assistance levels, rehabilitation, Trunk Impairment Scale

## Abstract

**Background:**

Achieving independent gait at discharge from an acute care hospital remains challenging, necessitating the identification of factors associated with different levels of gait assistance. Unlike previous studies, this study emphasizes the prognostic value of the Trunk Impairment Scale (TIS) when assessed within 48 h of stroke onset. Our findings highlight the predictive utility of early trunk function assessment in determining gait assistance levels at discharge and provide actionable insights for optimizing rehabilitation planning in acute care settings.

**Objective:**

This study is aimed at comparing gait assistance levels at discharge among patients with acute stroke and examining the association between gait assistance and trunk function.

**Methods:**

This prospective observational study included 115 stroke patients who were unable to achieve independent gait at discharge from an acute care hospital. Evaluations were conducted within 48 h of stroke onset and the day before discharge. Trunk function was assessed using the TIS, a practical bedside tool for evaluating core stability. Gait ability at discharge was classified as mild, moderate, or severe.

**Results:**

Significant differences in TIS scores were observed between the mild and moderate assistance groups (*p* < 0.05), mild and severe assistance groups (*p* < 0.001), and moderate and severe assistance groups (*p* < 0.01). The odds ratio (OR) for TIS scores was 1.53 (95% confidence interval [CI]: 1.06–2.20, *p* < 0.05) when comparing the mild and moderate assistance groups. For the moderate and severe assistance groups, the OR was 0.70 (95% CI: 0.53–0.92, *p* = 0.01). No significant associations were found for other variables.

**Conclusions:**

TIS score was strongly associated with gait assistance level, underscoring their predictive value for discharge gait ability. Early TIS assessments facilitate accurate prognostication and support the development of individualized rehabilitation plans, potentially improving functional outcomes in acute care settings.

## 1. Introduction

Although prior studies have explored trunk function several weeks after stroke onset, the immediate implications of early assessments on discharge outcomes remain underexplored. Trunk function has been widely studied using tools such as the Trunk Control Test (TCT) [1] and the Trunk Impairment Scale (TIS) developed by Verheyden et al. [2]. Both assessment tools have proven useful for evaluating trunk performance, particularly in postacute and chronic stroke settings [3–5]. However, the Verheyden version of the TIS, which evaluates both static and dynamic trunk function, requires more time and patient cooperation, which may limit its applicability in acute‐phase settings [1].

On the other hand, the TCT primarily assesses trunk function from a residual capacity perspective, focusing on ability rather than function. As a result, it measures trunk performance based on remaining capabilities rather than assessing functional aspects such as dynamic postural adjustments and coordination [2].

In contrast, the TIS developed by Fujiwara et al. [6] has been specifically designed for acute‐phase stroke patients. This version incorporates dynamic components of trunk function while ensuring simplicity and feasibility for bedside use in severely impaired patients. This study addresses this gap by utilizing the Fujiwara TIS to evaluate trunk function within 48 h of stroke onset, providing novel insights into its predictive value for discharge gait assistance levels.

Gait is crucial for achieving independence, and improving gait function is a key objective in stroke rehabilitation. However, many patients are unable to regain independent gait by discharge and must instead rely on assisted gait or wheelchairs. The extent of brain injury has been reported to influence gait recovery prognosis [7–9]. Furthermore, hemiplegia severity, basic motor skills, and activities of daily living (ADL) performance, trunk function, lower limb muscle strength, and balance have all been identified as key predictors of gait ability in stroke patients [5, 10, 11]. A study on hospital stay duration and outcomes in stroke patients, using the Medical Rehabilitation Uniform Data System, reported a reduction in hospital stay durations [12], potentially complicating the achievement of independent gait before discharge during the acute phase. Therefore, understanding gait status at discharge is crucial. However, most prior acute‐phase studies have examined outcomes several weeks after onset [3, 4, 13, 14], and few have longitudinally tracked outcomes from early onset through discharge. Studies that have integrated trunk function evaluation into stroke prognosis have primarily employed assessment methods focusing on sitting balance [1, 4, 5]. One possible reason for this is the lack of an established method to evaluate trunk dysfunction from a functional perspective in the early phase of stroke. In response, Fujiwara et al. developed the TIS to evaluate trunk performance from a functional viewpoint [6]. This study is aimed at assessing gait assistance levels at discharge in acute stroke patients and examining the association between assisted gait and trunk function using the TIS developed by Fujiwara et al. A clearer understanding of assisted gait is expected to provide valuable insights for optimizing rehabilitation strategies and implementing effective interventions at an early stage.

## 2. Materials and Methods

**Figure 1:**
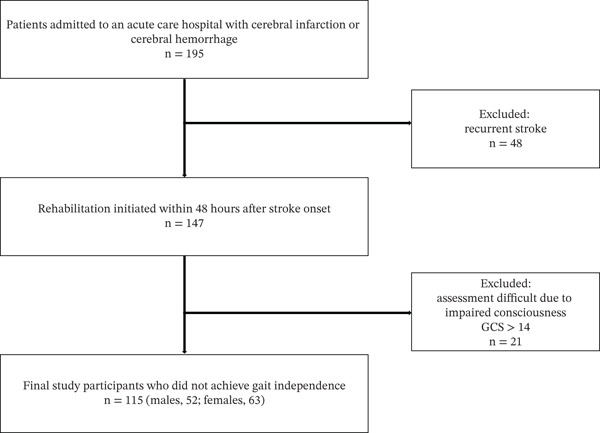
Inclusion criteria for patients in the present study.

This prospective observational study included 195 patients hospitalized for cerebral infarction or hemorrhage in an acute care facility between April 2020 and March 2022. The inclusion criterion was a first‐diagnosis of unilateral stroke, including both cerebral infarction and intracerebral hemorrhage, confirmed via brain computed tomography (CT) or magnetic resonance imaging (MRI). Rehabilitation was initiated within 48 h of stroke onset. Medical stability was ensured under physician supervision, and rehabilitation was initiated following stroke risk management protocols. Consciousness levels were classified based on the Glasgow Coma Scale (GCS), with eligible patients scoring ≥ 14, indicating they were awake and able to follow commands. Patients were excluded if they had impaired consciousness preventing clinical evaluation, had undergone surgery, experienced stroke deterioration, or did not survive. A total of 115 patients (52 males and 63 females) who did not regain independent gait were included in this study (Figure 1). The median age of participants was 80 years (interquartile range (IQR) 71.0–87.0 years), and the median length of stay (LOS) was 23 days (IQR 19.0–33.0). Sixty patients had right‐sided impairment, and 55 patients had left‐sided impairment (Table 1). All participants received a detailed explanation of the study objectives, and written informed consent was obtained. For those unable to provide a signature, consent was obtained from an authorized representative, such as a family member. This study was approved by the Ethics Board of Shioda Hospital, Chiba, Japan (Approval Number: 201911). All procedures were conducted in accordance with the Declaration of Helsinki.

**Table 1:** Participant characteristics.

Survey item	Total cases (*n* = 115)	

Number of patients (male/female)	115 (52/63)	
Age (years old, median, IQR)	80 (71–87)	
Length of stay (day, median, IQR)	23 (19–33)	
Cerebral hemorrhage	37	
Cerebral infarction	78	
Number of paralytic side (Rt/Lt)	60/55	

	Hospitalization	Discharge from hospital

NIHSS (median, IQR)	11 (6–15.5)	6 (3–11)
TIS (median, IQR)	6 (3–11)	14 (10–17)
SIAS‐M (median, IQR)	8 (4–14)	14 (7–18)

*Note:* mean ± SD.

Abbreviations: NIHSS, National Institutes of Health Stroke Scale; SIAS‐M, Stroke Impairment Assessment Set‐Motor; TIS, Trunk Impairment Scale.

### 2.1. Methods

Key data, including diagnosis, affected side, age, sex, and LOS were collected. The sample size was determined based on the number of eligible patients hospitalized during the study period. No prior sample size calculation was conducted. The severity of the stroke was measured using the National Institutes of Health Stroke Scale (NIHSS) [15]. Motor function on the affected side was evaluated using the movement components of the stroke impairment assessment set (SIAS‐M) [16]. Trunk function was assessed using the TIS developed by Fujiwara et al. [6]. The TIS consists of seven items, including awareness of trunk verticality, rotational strength on both the affected and unaffected sides, righting reactions, abdominal muscle strength, and the upright posture test from the SIAS trunk function section. The total TIS score ranged from 0 to 21 points. ADLs were assessed at discharge from the acute care hospital using the Functional Independence Measure (FIM) [17]. Mobility classification was based on the primary mode of transportation at discharge, either gait or wheelchair use. For patients with assisted gait, classification was determined using both the FIM locomotion gait scores and actual walking ability observed during rehabilitation sessions.

• Light assistance group (FIM = 4 points): Patients could walk with minimal physical contact, requiring only occasional steadying assistance.
• Moderate assistance group (FIM = 3 points): Patients required continuous manual support from a therapist but were able to take at least several steps independently.
• Heavy assistance group (FIM = 1–2 points): Patients were unable to initiate stepping movements independently and required full weight‐bearing support or a wheelchair.

The initial evaluation was conducted within 48 h of stroke onset, and the final evaluation took place the day before discharge. All assessments were performed by the same examiner to ensure consistency.

### 2.2. Statistical Analysis

The Shapiro–Wilk test was performed before each analysis to assess whether each variable followed a normal distribution. Missing data were excluded from the analysis, as no imputation methods were described. Additionally, patients lost to follow‐up were excluded from the study, as they were unable to complete the evaluations. No sensitivity analyses were conducted, as the study focused on predefined variables and fixed time points. Spearman′s rank correlation analysis was conducted to identify variables with an absolute correlation coefficient (r) ≥ 0.9, and to examine the relationship between gait assistance levels and each measurement item. Variables with a variance inflation factor (VIF) ≥ 10 were also analyzed to assess multicollinearity. For gait assistance level at discharge, participants were classified into three groups: light assistance, moderate assistance, and heavy assistance. The Kruskal–Wallis test was applied to each evaluation item. For categorical variables, the chi‐square (*χ* ^2^) test was used. For items showing significant differences, post hoc multiple comparisons were performed using the Steel–Dwass method. To analyze the factors affecting gait assistance levels, multinomial logistic regression analysis was conducted, with gait assistance level as the dependent variable and key predictor variables including stroke type, hospital stay duration, NIHSS, TIS, and SIAS‐M. All statistical analyses were performed using IBM SPSS Statistics software (Version 29), with statistical significance set at *p* < 0.05.

## 3. Results

### 3.1. Participants Selection Process and Nonparticipation Reasons

This study initially included 195 patients who were admitted to an acute care hospital with a diagnosis of either cerebral infarction or cerebral hemorrhage. After applying the eligibility criteria, 48 patients were excluded due to recurrent stroke, having undergone surgery, deterioration of stroke condition, or mortality. Of the remaining 147 patients who initiated rehabilitation within 48 h of stroke onset, 32 were further excluded due to assessment difficulties caused by impaired consciousness (GCS ≥ 14). Thus, the final analysis comprised 115 participants (52 males and 63 females) who had not attained independent gait.

### 3.2. Comparison of Attributes and Physical Function Evaluations at Discharge

**Figure 2:**
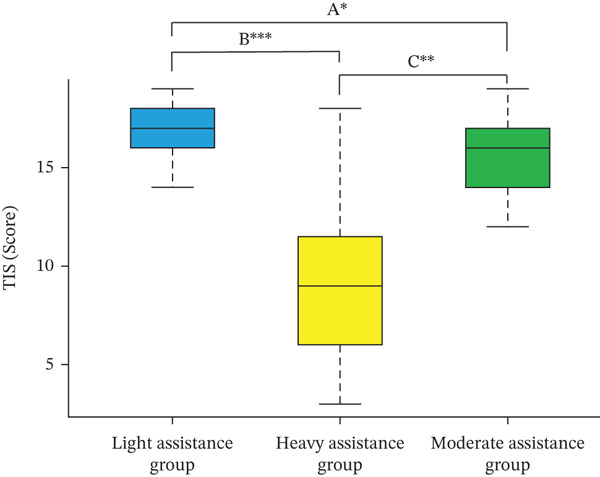
Multiple comparison results based on TIS scores using the Steel–Dwass method. (A) The light assistance group versus the moderate assistance group, ∗*p* < 0.05. (B) The light assistance group versus the heavy assistance group, ∗∗∗*p* < 0.001. (C) The heavy assistance group versus the moderate assistance group, ∗∗*p* < 0.01.

Table 2 presents the attributes and evaluation results at discharge, categorized by gait assistance level, along with comparative analyses. Analysis of variance (ANOVA) revealed significant differences in hospital stay duration (*p* < 0.01), disease type (*p* < 0.05), NIHSS score (*p* < 0.001), TIS score (*p* < 0.001), and SIAS‐M score (*p* < 0.001). For items with significant differences, the results of post hoc multiple comparisons were as follows:

• Length of hospital stay: A significant difference was observed between the moderate and heavy assistance groups (*p* < 0.01).
• Disease type: A significant difference was identified between the light and heavy assistance groups (*p* < 0.05).
• NIHSS score: Significant differences were found between the light and moderate assistance groups (*p* < 0.001) and between the moderate and heavy assistance groups (*p* < 0.01). Notably, patients with an NIHSS score of 8 were classified into the moderate assistance group rather than the heavy assistance group. This classification was based on their observed ability to initiate stepping movements with manual support, despite moderate limb impairments. In contrast, patients in the heavy assistance group demonstrated complete dependency on external support and an inability to initiate stepping.
• TIS score: Significant differences were detected between the light and moderate assistance groups (*p* < 0.05), between the light and heavy assistance groups (*p* < 0.001), and between the moderate and heavy assistance groups (*p* < 0.001) (Figure 2).
• SIAS‐M score: Significant differences were observed between the light and moderate assistance groups (*p* < 0.05), between the light and heavy assistance groups (*p* < 0.001), and between the moderate and heavy assistance groups (*p* < 0.001).

**Table 2:** Results from a comparison of three groups: The independent gait group.

Survey item	Light assistance group (*n* = 30)	Moderate assistance group (*n* = 24)	Heavy assistance group (*n* = 61)	*p*	Steel–Dwass									
Diagnosis name														
Number of patients (male/female)	30 (14/16)	24 (15/9)	61 (23/38)	0.12			_							
Age	78.0 (66.0–87.0)	80.0 (75.0–85.0)	80.0 (73.0–87.0)	0.69			_							
Length of stay (day, median, IQR)	20.0 (18.0–25.0)	20.0 (15.0–25.0)	26.0 (20.0–35.0)	< 0.01		C	^∗∗^							
Cerebral hemorrhage	4	7	26	0.02		B	^∗^							
Cerebral infarction	26	17	35											
Number of paralytic side (Rt/Lt)	16/14	12/12	32/29	0.97					_					
NIHSS (median, IQR)	3.0 (2.0–5.0)	3.0 (3.0–8.0)	9.0 (6.0–13.0)	< 0.001		A	^∗∗∗^		C	^∗∗^				
TIS (median, IQR)	17.0 (16.0–18.0)	16.0 (14.0–17.0)	10.0 (6.0–14.0)	< 0.001		A	^∗^		B	^∗∗∗^		C	^∗∗∗^	
SIAS‐M (median, IQR)	18.0 (15.0–20.0)	17.0 (14.0–18.0)	7.5 (5.0–13.0)	< 0.001		B	^∗∗∗^		C	^∗∗∗^				

*Note:* mean ± SD. Multiple comparisons. A: light assistance group and moderate assistance group. B: light assistance group and heavy assistance group. C: moderate assistance group and heavy assistance group.

Abbreviations: NIHSS, National Institutes of Health Stroke Scale; SIAS‐M, Stroke Impairment Assessment Set‐Motor; TIS, Trunk Impairment Scale.

∗*p* < 0.05.

∗∗*p* < 0.01.

∗∗∗*p* < 0.001.

### 3.3. Relationship Between Gait Assistance Level at Discharge and Attributes/Evaluation Items

Gait assistance level was significantly correlated with hospital stay (*r* = 0.30, *p* < 0.01), disease type (*r* = 0.26, *p* < 0.01), NIHSS score (*r* = 0.57, *p* < 0.001), TIS score (*r* = −0.66, *p* < 0.001), and SIAS‐M score (*r* = −0.625, *p* < 0.001). No significant correlations were observed for the remaining variables.

### 3.4. Results of the Multinomial Logistic Regression With Gait Assistance Level as the Outcome Variable

Multinomial logistic regression analysis identified the TIS score as a significant predictor for both the light and heavy assistance groups compared with the moderate assistance group (Tables 3 and 4). No statistically significant risk ratios were observed for the remaining variables.

**Table 3:** Results of the multinomial regression analysis with gait assistance level as the target variable (Risk ratio of the light assistance group to the moderate assistance group).

Regression coefficient	OR	95% CI	*p*
Diagnosis	1.06	0.98–1.14	0.19
Length of stay	1.06	0.98–1.14	0.16
NIHSS	0.98	0.77–1.29	0.98
TIS	1.54	1.06–2.24	0.02
SIAS‐M	1.04	0.87–1.25	0.68

Abbreviations: CI, confidence interval; NIHSS, National Institutes of Health Stroke Scale; OR, odds ratio; SIAS‐M, Stroke Impairment Assessment Set‐Motor; TIS, Trunk Impairment Scale.

**Table 4:** Results from the multinomial logistic regression analysis with gait assistance level as the objective variable (Risk ratio of the moderate assistance group to the heavy assistance group).

Regression coefficient	OR	95% CI	*p*
Diagnosis	1.09	0.30–4.02	0.90
Length of stay	1.03	0.96–1.11	0.46
NIHSS	0.92	0.74–1.14	0.46
TIS	0.70	0.53–0.92	0.01
SIAS‐M	0.85	0.72–1.01	0.06

Abbreviations: CI, confidence interval; NIHSS, National Institutes of Health Stroke Scale; OR, odds ratio; SIAS‐M, Stroke Impairment Assessment Set‐Motor; TIS, Trunk Impairment Scale.

## 4. Discussion

The early implementation of the TIS not only facilitates the timely identification of patients in need of intensive rehabilitation but also enables the customization of interventions based on individual needs. This approach can enhance resource allocation in acute care settings and, ultimately, improve patient recovery trajectories.

In recent years, early rehabilitation interventions following stroke onset have significantly impacted patients′ prognosis [18–22]. In particular, to promote the early acquisition of walking ability, it is essential to identify key prognostic factors and implement rehabilitation strategies accordingly [23–25]. Many stroke patients require gait assistance due to the risk of falling, and rehabilitation plays a crucial role in maximizing functional independence [26]. However, despite the continued reduction in the LOS in acute care hospitals, achieving independent gait at discharge remains a significant challenge. Discharge criteria for stroke rehabilitation inpatients encompass multiple factors, including functional status, socioeconomic considerations, medical conditions, family support, and architectural barriers at home. Additionally, gait ability is considered a key determinant of discharge destination. This study is aimed at comparing gait assistance levels at discharge among patients with acute stroke and examining the relationship between assisted gait and trunk function using the TIS developed by Fujiwara et al. as a contributing factor to assisted gait. Trunk muscles are bilaterally innervated, making them less susceptible to stroke‐related impairments compared with the upper and lower limbs [27]. However, the delayed recovery of trunk function can lead to postural instability, increased compensatory upper limb use, and asymmetric weight distribution. As a result, proper weight shifting and center‐of‐gravity movement during gait initiation become difficult, leading to a decrease in propulsive force. Consequently, gait stability declines, increasing the likelihood of requiring gait assistance [28–30]. The control of trunk function involves both the lateral and medial motor control systems. The lateral motor control system, including the lateral corticospinal tract and the rubrospinal tract, is involved in regulating distal limb movements, fine motor control, and sensory input modulation. On the other hand, the medial motor control system, including the corticoreticulospinal tract, tectospinal tract, lateral vestibulospinal tract, and anterior corticospinal tract, plays a crucial role in stabilizing trunk muscles, postural reflexes, balance function, and gait control [31, 32]. In particular, the greater the impairment of the medial motor control system, the more it leads to reduced righting reactions and deficits in anticipatory postural adjustments (APA), resulting in compromised stability during gait initiation and directional changes. As a result, proper center‐of‐gravity movement is hindered, leading to decreased dynamic stability and an increased risk of falls, ultimately raising the need for gait assistance. Additionally, previous studies using transcranial magnetic stimulation (TMS) have suggested that the recovery of trunk function in hemiparetic patients involves ipsilateral pathways from the nonlesioned hemisphere [33]. This suggests that facilitating trunk function not only on the affected side but also on the nonaffected side may help compensate for impaired postural control on the paretic side, thereby improving balance maintenance during gait. In this study, patients with impaired trunk function tended to require higher levels of gait assistance at discharge. Specifically, it was confirmed that patients with lower TIS scores were more likely to require gait assistance. This is considered to be due to the dysfunction of the medial motor control system, which impairs dynamic stability during gait. In the future, to reduce the need for gait assistance, early trunk function assessment and appropriate intervention strategies from the acute phase will be essential. In particular, further investigation is needed on the effectiveness of rehabilitation focusing on activating trunk function on the nonaffected side. Previous studies on gait prognosis have utilized the Berg Balance Scale to assess balance ability, the Fugl–Meyer assessment to evaluate lower limb motor paralysis, and both the TCT and the TIS developed by Verheyden et al. for assessing trunk function [10, 11, 34]. Prognostic evaluations related to gait are commonly conducted 2 weeks after onset, with many studies focusing on assessments at 3 and 6 months postonset [3, 4, 10–14]. However, to date, no studies have examined the relationship between factors assessed within 48 h of stroke onset and gait ability at discharge, approximately 3 weeks later, as investigated in this study.

ANOVA comparing the three gait assistance groups at discharge identified significant differences in the length of hospital stay, disease type, and NIHSS, TIS, and SIAS‐M scores. Post hoc multiple comparisons for these five variables revealed a significant difference in the length of hospital stay between patients requiring moderate and heavy assistance, indicating a tendency for longer hospital stays as the level of assistance increases. Patients requiring heavy assistance typically have multiple impairments or complications that complicate rehabilitation and treatment, which may contribute to prolonged hospital stay. This finding suggests that the level of assistance required by patients significantly influences hospital stay duration, highlighting the need to consider assistance level when designing appropriate rehabilitation and care plans.

A statistically significant difference in SIAS‐M scores was observed among the gait assistance groups. Specifically, significant differences in SIAS‐M scores were found between the light and heavy assistance groups as well as between the moderate and heavy assistance groups. This suggests that as the level of assistance increased, stroke‐related motor dysfunction tended to worsen. Additionally, TIS scores differed significantly across all group comparisons, suggesting that variations in trunk function recovery exist even among different gait assistance levels following stroke. In particular, the heavy assistance group exhibited lower TIS scores than the other groups, indicating that these patients experienced slower recovery of trunk function compared with requiring light or moderate assistance. Consistent with previous studies, this finding supports the notion that trunk function recovery serves as a predictor of early motor dysfunction severity [35].

A significant association was observed between gait assistance level at discharge and functional and motor function evaluations, specifically NIHSS, TIS, and SIAS‐M scores. The classification criteria for gait assistance were carefully determined based on both FIM scores and observed walking ability. Although NIHSS scores provide a useful measure of neurological impairment, gait assistance needs are influenced by multiple factors beyond the NIHSS score alone, including balance control, compensatory strategies, and rehabilitation progress.

In particular, patients with an NIHSS score of 8 demonstrated moderate impairments but were able to participate in assisted gait training, justifying their classification in the moderate assistance group. These findings suggest that patients with better recovery of trunk and motor functions tend to require lower levels of gait assistance, which is consistent with previous studies [11].

In the multinomial logistic regression analysis, with the moderate assistance group as the reference, a significant difference in the TIS scores was observed in both the light and heavy assistance groups. For each 1‐point increase in the TIS score, the odds of requiring light rather than moderate assistance increased by 1.53 times, whereas the odds of requiring heavy rather than moderate assistance decreased by 0.70 times. Higher TIS scores indicate a lower likelihood of requiring moderate gait assistance in stroke patients, as well as a reduced probability of needing heavy assistance. In other words, greater trunk function (higher TIS score) is associated with less reliance on gait assistance. Trunk function plays a crucial role in postural control, balance, gait, and ADL. Effective trunk movement control is essential for coordinating hand and foot movements and maintaining balance. Furthermore, proper trunk control enhances functional mobility and gait following stroke [36, 37]. The findings of this study underscore the value of the TIS in acute‐phase rehabilitation planning. By utilizing TIS scores assessed within 48 h of stroke onset, clinicians can predict patients′ gait assistance needs at discharge and tailor rehabilitation strategies accordingly. For instance, patients with lower TIS scores may benefit from intensive trunk stabilization exercises to improve postural control and balance, whereas those with higher TIS scores may transition more quickly to gait training.

This approach facilitates the development of individualized rehabilitation plans tailored to each patient′s functional prognosis, thereby optimizing resource allocation and potentially reducing hospital stays. Additionally, the simplicity and feasibility of bedside TIS assessments make them a practical tool for routine use, even in severely impaired patients. These applications underscore the importance of early trunk function assessment in improving clinical outcomes and enhancing the overall quality of care in acute stroke settings. Therefore, proper trunk control serves as a key indicator of gait function and functional recovery in stroke patients. When combined with other physical function evaluation scales, trunk control enables a more comprehensive assessment of a patient′s condition, contributing to the development of effective rehabilitation programs. Furthermore, unlike other trunk function assessments, the TIS is simple to administer at the patient′s bedside, even for individuals with difficulty maintaining a seated position, making it highly practical in clinical settings. This makes it a valuable tool for promoting the early initiation of rehabilitation [6, 18, 22, 38]. In future clinical practice, the integration of multifaceted evaluations, including the TIS, will be essential in early‐stage rehabilitation planning to develop individualized rehabilitation strategies. This approach is expected to maximize functional recovery and improve the quality of life following discharge.

This study had a few limitations. First, the frequency and content of rehabilitation varied among individuals, which may have contributed to differences in gait improvement. Additionally, factors such as patients′ activities outside of rehabilitation, the nature of their training, and the expertise of their instructors may have influenced the findings. Second, this study was conducted in a single facility and focused exclusively on patients with acute stroke, limiting the generalizability of the results. As a result, it remains uncertain whether these findings can be replicated in different clinical settings or with alternative rehabilitation programs. Third, this study did not account for potential variations in evaluator assessments, which represents a limitation. Ensuring consistency among evaluators is essential for future research to improve reliability. Fourth, the reliability of the TIS used in this study, as well as its validity in predicting assisted gait levels, was not independently verified within our dataset. Although the TIS developed by Fujiwara et al. has demonstrated adequate psychometric properties in previous studies, including reliability and responsiveness, these aspects were not reassessed here. Additionally, a direct comparison with the original version of the TIS developed by Verheyden et al. was not conducted, leaving the relative significance of the selected version unexamined. Future studies should consider including comparative evaluations of different TIS versions to validate the generalizability of the findings. Furthermore, this study focused solely on patients with acute stroke and did not assess changes or recovery in gait ability after discharge. Future research should incorporate long‐term monitoring studies to evaluate the maintenance and improvement of gait function following discharge, particularly in home based settings.

## 5. Conclusions

This study found that trunk function, as measured using the TIS, was strongly associated with gait assistance levels at discharge in patients with acute stroke. These findings suggest that patients with stronger trunk function and motor abilities are likely to require lower levels of gait assistance. Specifically, the TIS serves as an effective predictor of gait ability at discharge, with higher scores indicating a reduced likelihood of requiring moderate or heavy gait assistance. Moreover, the TIS offers practical advantages in clinical settings, as it can be easily administered at the bedside, even for patients who have difficulty maintaining a seated position, making it a valuable tool for early rehabilitation. Incorporating early and multifaceted evaluations, including the TIS, into individualized rehabilitation plans is essential for maximizing functional recovery and enhancing patients′ quality of life following discharge.

## Funding

No funding was received for this manuscript.

## Conflicts of Interest

The authors declare no conflicts of interest.

## Data Availability

The data that support the findings of this study are available from the corresponding author upon reasonable request.
